# lazar: a modular predictive toxicology framework

**DOI:** 10.3389/fphar.2013.00038

**Published:** 2013-04-09

**Authors:** Andreas Maunz, Martin Gütlein, Micha Rautenberg, David Vorgrimmler, Denis Gebele, Christoph Helma

**Affiliations:** ^1^Institute for Physics, Albert-Ludwigs-Universität FreiburgFreiburg, Germany; ^2^in silico toxicology gmbhBasel, Switzerland

**Keywords:** predictive toxicology, QSAR, *in silico*, semantic web, read across

## Abstract

lazar (lazy structure–activity relationships) is a modular framework for predictive toxicology. Similar to the *read across* procedure in toxicological risk assessment, lazar creates local QSAR (quantitative structure–activity relationship) models for each compound to be predicted. Model developers can choose between a large variety of algorithms for descriptor calculation and selection, chemical similarity indices, and model building. This paper presents a high level description of the lazar framework and discusses the performance of example classification and regression models.

## INTRODUCTION

Computer-based (*in silico*) predictions are gaining acceptance in toxicological risk assessment, but there is still a lot of reservation toward *in silico* methods, especially from toxicologists with a biological or medical background. Apart from obvious barriers between the involved disciplines, we attribute this reservation to a variety of scientific, technical, and social factors:

### SCIENTIFIC LIMITATIONS

- Limited capability of some quantitative structure–activity relationship (QSAR) algorithms (e.g., linear regression) to handle complex relationships
- Missing, improper, ambiguous, or poorly reproducible definitions of applicability domains
- Improper application of validation procedures, ignorance of applicability domains^1^
- Poor validation of applicability domain concepts
- Poor consideration of biological mechanisms
- Irreproducible results, because proprietary algorithms are not disclosed

### TECHNICAL LIMITATIONS

- Hard to use and unintuitive software
- Standalone solutions with poor integration of external databases, ontologies etc.

### SOCIAL LIMITATIONS

- Insufficient translation of statistics/data mining/QSAR concepts into toxicological terminology
- Poor understanding of the significance of validation results^1^
- Poor and/or too technical documentation of algorithms, which is hard to understand for non-computer scientists

We have developed lazar (shortcut for lazy structure–activity relationships) approximately 5~years ago in order to address some of these shortcomings and to fulfill the requirements of the Organisation for Economic Co-operation and Development (OECD) principles for QSAR validation (Organisation for Economic Co-operation and Development [OECD], 2004b). In the meantime it has undergone several revisions and rewrites and ended up as a completely modular framework for predictive toxicology, based on the OpenTox (Hardy et al., 2010) framework. This paper documents the main modifications of lazar, implementation details, new algorithms, and experiments performed since the original lazar publications (Helma, 2006; Maunz and Helma, 2008). It is intended as a high level overview for readers without a background in computer science or data mining. Readers interested in algorithmic details should consult the original literature cited in the references, and the source code documentation at Github^2^.

## METHODS

### OVERVIEW

The main objective of lazar is to provide a generic tool for the prediction of complex toxicological endpoints, like carcinogenicity, long-term, and reproductive toxicity. As these endpoints involve a huge number of complex (and probably unknown) biological mechanisms, lazar does not intend to model all involved biological processes (as in molecular modeling or various systems biology approaches), but follows a *data driven* approach.

lazar uses data mining algorithms to derive predictions for untested compounds from experimental training data. Any dataset with chemical structures and biological activities can be used as training data. This makes lazar a generic prediction algorithm for any biological endpoint with sufficient experimental data.

At present, lazar does not consider chemical, biological, or toxicological expert knowledge, but derives computational models from statistical criteria. Such an approach has the distinct advantage that incomplete, wrong, or incorrectly formulated background knowledge cannot affect predictions, because they are based on objective, traceable, and reproducible statistical criteria^3^.

Although lazar does not use explicit background knowledge for predictions, it was created with an intent to support mechanistic-based risk assessment. For this purpose, rationales for predictions are presented together with a *hypothesis* about possible biological mechanisms that is based on statistically significant properties of the underlying data^4^. As both, predictions and mechanisms, are statistically derived (not causally or mechanistically), the toxicological expert is a key part of the process. He should review and interpret the output in order to identify, e.g., training data errors, chance correlations, systematic problems, or findings that contradict with current knowledge and discard results if necessary^5^.

**FIGURE 1:**
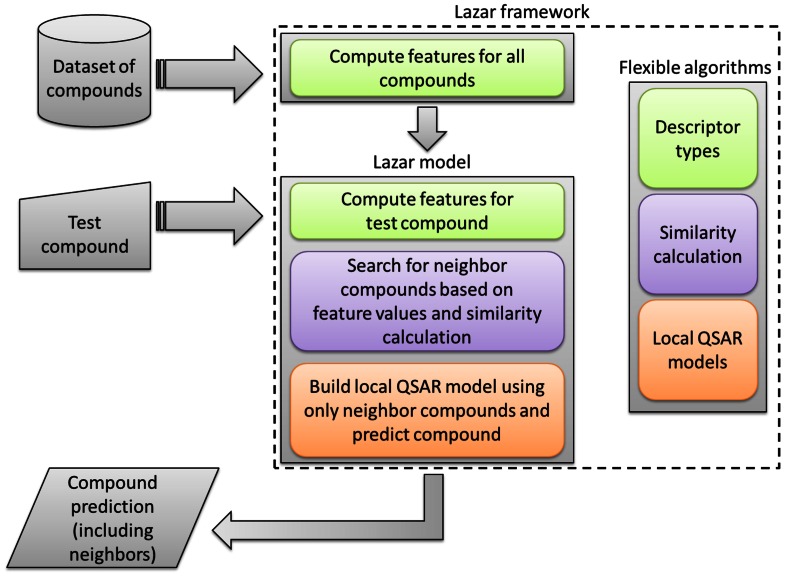
**The workflow of the lazar framework, with regard to the configurable algorithms for descriptor calculation, chemical similarity calculation, and local QSAR models**.

In contrast to most machine learning and QSAR methods, which create a global prediction model from all training data, lazar uses local QSAR models, similar to the *read across* procedure (**Figure 1**). To obtain a prediction for a given query compound lazar

- identifies *similar* compounds in the training data (*neighbors*)
- creates a local prediction model (based on experimental activities of *neighbors*)
- uses the local model to predict properties of the query compound

We have shown experimentally (Helma, 2006; Maunz and Helma, 2008) that this procedure gives superior results compared to global models, which is also in consensus with the commonly accepted notion in the QSAR community that local QSAR models provide results superior to global QSAR models (Guha et al., 2006). For this reason, the core prediction scheme remains unaltered in lazar, but considerable flexibility arises from the selection of algorithms for

- descriptor calculation
- chemical similarity calculation
- local QSAR models

lazar is able to utilize OpenTox compatible algorithm implementations. Within the scope of the collaborative EU project OpenTox, a unified interface for an interoperable predictive toxicology framework was defined, and several applications and services have been created. The available OpenTox implementations give us access to many chemoinformatics and data mining algorithms implemented in open source projects like Chemistry Development Kit (CDK; Steinbeck et al., 2006), OpenBabel (O’Boyle et al., 2011), R (R Core Team, 2012), and WEKA (Hall et al., 2009). In addition we have implemented novel algorithms for substructure mining and similarity calculations, which are described below.

lazar fills a niche between specialized toxicity prediction tools^6^, which rely mostly on pre-built models and general purpose statistical and data mining tools (like R or WEKA) which lack chemoinformatics algorithms for the predictive toxicology domain and are frequently hart to use for non-experts. lazar streamlines the model building and validation process and creates standalone prediction models that can be used without prior processing of input data (e.g., external descriptor calculation).

### ALGORITHMS

Several types of algorithms ensure the flexibility of the lazar system. **Figure 1** shows the integration of these algorithms into the workflow.

#### Similarity

Although the concept of chemical similarity is very intuitive at a first glance, there is no global *similarity* property intrinsic to chemical structures (Raymond and Willett, 2002). Instead, there are many ways to define chemical similarity, and each of them may serve different purposes.

***Structural similarity*.** The similarity between structures is the most frequently used chemical similarity concept. Although visually obvious for the trained eye of a chemist, it is far from straightforward to define structural similarity formally. A few methods can work with structure graphs directly, but they are too computationally expensive for practical purposes (e.g., database searches). Most practical methods require the decomposition of structures into a set of distinct substructures (fingerprints). While standard chemoinformatics libraries provide methods based on predefined fingerprints (Raymond and Willett, 2002), we have developed methods that allow us to mine efficiently for relevant substructures (see Substructure Mining) and use them to determine *activity specific* similarities. Technically, most structural similarity indices work with either with binary (i.e., true/false) classifications, indicating the presence of a given substructure in a compound, or consist of substructure frequency counts.

***Property similarity*.** It can be argued that the biological activity of a compound is not determined by its structure *per se*, but by its physico-chemical properties. However, these are in turn determined by chemical structure. Physico-chemical properties can be determined either experimentally, or calculated from chemical structure. Although many similarity indices from the literature combine physico-chemical properties and substructures in a single index, we prefer to keep both concepts separated. Technically, we have to work with numerical values instead of nominal class values.

***Biological similarity*.** The similarity of compounds can be also determined by their biological behavior. Although it is frequently (silently) assumed that similar structures exhibit similar biological behavior, every pharmacology and toxicology textbook provides examples where a small modification of the chemical structure causes a big difference in biological effects. It is therefore useful to define biological similarities in addition to structural and property similarities. Descriptors for biological similarity can originate, for example, from high throughput assays [as in the ToxCast (Dix et al., 2007) exercise] and may consist of quantitative assay results, affected targets, or pathways, among others. Technically, they will have to work with numerical values as well as binary classifications. It is also essential that the similarity index handles missing values gracefully.

***Activity specific similarities*.** The calculation of similarity indices may require large lists of descriptors, most of them unrelated to the endpoint under investigation. In the case of structural similarity our intention is to compare only *biologically active* parts of the molecule, and ignore the inert parts. For this purpose we have defined *activity specific similarities*, which weight the contribution of each descriptor by its correlation with a given endpoint. Weights are determined by simple statistical tests (e.g., Chi-square test), and descriptors below a predefined threshold are discarded.

We were able to show that prediction accuracies can be improved significantly (Helma, 2006; Maunz and Helma, 2008) with activity specific similarities. This procedure yields also lists of *relevant* descriptors as an important byproduct, which can be useful to indicate possible biological mechanisms, or provide directions for designing safer compounds.

***Similarity indices***. Two implementations exist, depending on descriptor type.

- Substructures: Employs a weighted Tanimoto index to determine neighbors to the query structure and derive a prediction from them. The Tanimoto index is essentially a set kernel (Gärtner, 2006). The related Tanimoto index is one of the most useful chemical similarity indices, as shown by Willet and colleagues (Holliday et al., 2002). It encodes presence or absence of substructures in molecules, or the number of times substructures occur in molecules.
- Physico-chemical properties: The features are preprocessed using a singular value decomposition (SVD). This has many desirable effects, e.g., normalization of the feature value range, selection of the most expressive features, and redundancy reduction. Subsequently, the distance between two compounds is computed using cosine similarity, by measuring the angle between the feature value vectors. In natural language processing, this approach is known as Latent Semantic Indexing (Berry et al., 1995). The algorithm uses the Golub–Reinsch SVD algorithm (Galassi et al., 2009).

lazar provides a confidence value with every prediction, ranging between 0 and 1, based on the mean neighbor similarity.

#### Descriptor calculation

***Substructure mining*.** Substructure mining algorithms often produce huge sets of redundant chemical fragments with the same biochemical relevance (e.g., substructures that differ only by a few carbon atoms). Since experts cannot draw any conclusions from a vast amount of very similar substructures, it has been argued that uncompressed results would require post-processing (Chi et al., 2004; Huan et al., 2004; Schietgat et al., 2011), in order to find meaningful patterns. Similarly, a high-dimensional pattern space prevents machine learning methods from obtaining meaningful models (Al Hasan et al., 2007).

Backbone Refinement Class Mining (Maunz et al., 2011) and LAST-PM (Maunz et al., 2010) are two algorithmic approaches to mining compact sets of descriptors in the search space of chemical structure graphs, creating compressed and elaborate representations of chemical structure. Both methods combine feature generation and feature selection into one step.

Backbone Refinement Class Mining (BBRC) creates a sparse selection from the search space of frequent and significant subtrees, based on structural and statistical constraints. It has very high compression potential, which has been shown theoretically (Maunz et al., 2011). Empirical results confirmed the compression results in practice, while retaining good database coverage. Moreover, it has been shown that the structural constraints produce structurally diverse features with low co-occurrence rates. BBRC descriptors compare favorable to other compressed representations in the context of classification models.

Latent Structure Pattern Mining (LAST-PM) repeatedly combines related substructures into a weighted edge graph and mines elaborate patterns from this graph. The elaborate patterns differ in two aspects from basic substructures. First, the process superimposes the substructures, and substructures may differ in size. This yields different weights for the constituent nodes and edges (i.e., atoms and bonds). Heavy components (in terms of the weights) are extracted from the weighted edge graph by SVD, and the ambiguities are resolved by logical “OR” operations. It also generates ambiguities (e.g., oxygen or nitrogen at a certain position), since substructures may be conflicting, i.e., node and edge labels may differ at certain positions. The procedure yields a tightly condensed representation of the dataset. The resulting chemical fragments are expressed in a chemical fragment query language (SMARTS), preserving the ambiguities. They are interpretable for chemical experts.

**FIGURE 2:**
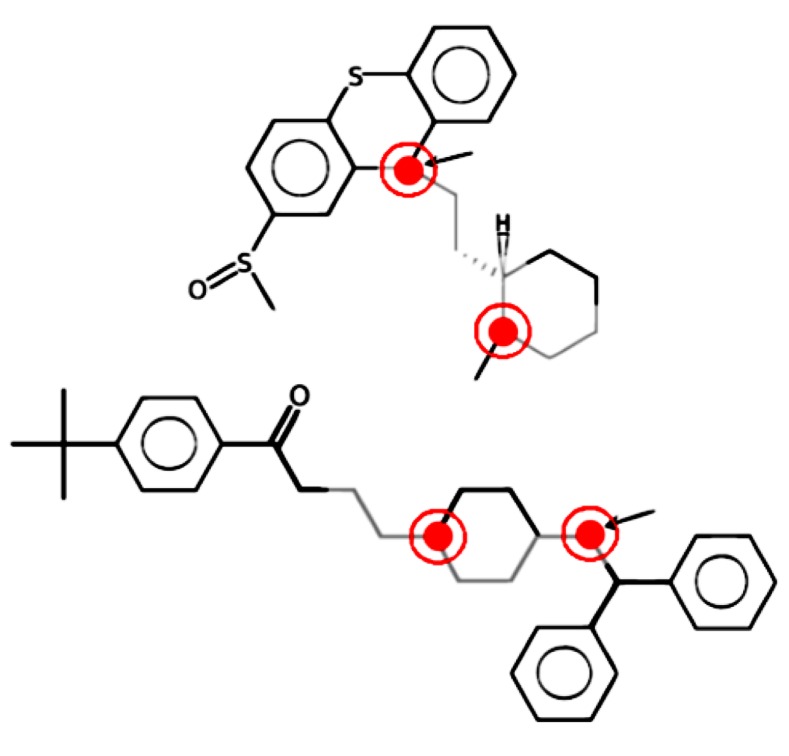
**A gray fragment with atom ambiguity, inducing polarity at the marked positions**.

As an example, in **Figure 2**, LAST-PM, instead of returning a set of similar fragments to the user, aligns the structure graphs and extracts a common motif. It is the gray fragment with two polarity inducing positions, marked red. The fragment is not identical in both molecules, but has an ambiguous position that abstracts from differences not influencing the toxicological behavior (the arrow-marked atom, which may be oxygen or nitrogen).

In classification tasks with either nearest-neighbor or support vector machine (SVM) models, the accuracy of (models based on) BBRC descriptors was on par with the complete set of frequent and significant subtrees, but significantly better than that of other compressed representations. LAST-PM descriptors performed even significantly better than the complete set from which they were derived. They also outperformed BBRC descriptors and highly optimized physico-chemical descriptor models from the literature in the classification of compounds for complex biological endpoints (Maunz et al., 2010). Both algorithms perform substructure selection with regard to the endpoint under investigation, and calculate substructure associations to the endpoint in the form of *p*-values.

***Physico-chemical properties*.** lazar utilizes open source chemoinformatics libraries to calculate a range of physico-chemical descriptors. Furthermore, other existing OpenTox (Hardy et al., 2010) compliant descriptor calculation services can be queried. Categories were formed for the available chemical descriptors (with a selection of descriptors):

**Constitutional:** largest chain, aromatic bonds count, longest aliphatic chain, rule of five, atom count, XLogP, ALOGP, aromatic atoms count, Mannhold LogP, bond count, rotatable bonds count, largest Pi system.

**Electronic:** APol, BPol, H-bond acceptor count, H-bond donor count, charged partial surface area descriptors (CPSA).

**Geometrical:** geometrical diameter, geometrical radius, gravitational index, length over breadth, moments of inertia.

**Topological:** Chi Path, fragment complexity, Kier–Hall Smarts, Kappa Shape Indices, Petitjean Number, autocorrelation mass, VAdjMa, Chi Path Cluster, Wiener Numbers, Autocorrelation Polarizability, carbon types, eccentric connectivity index, Chi Chain, MDE, Petitjean shape index, TPSA, Chi cluster, Zagreb index, autocorrelation charge.

**Hybrid:** Burden–CAS–University of Texas (BCUT) descriptor, weighted holistic invariant molecular (WHIM) descriptor.

In total, lazar can be used to generate more than 300 different, numerically unconstrained descriptors. In its current implementation, it is able to calculate all of them on its own.

***Measured properties***. In addition to calculated properties lazar can utilize experimental measurements (e.g., of physico-chemical properties or results from high-throughput assays) to characterize compounds. This allows us to encode *biological similarities* (e.g., in respect to affected targets or pathways) and to apply the lazar framework to compounds without well defined chemical structures like nano particles.

#### Learning algorithms

lazar uses a weighted majority voting scheme for classification, or SVM formulations for both classification and regression problems (numerical predictions). For the latter, either the Tanimoto kernel or the Gaussian radial basis function kernel is available. In any case, lazar builds a dedicated model for any single prediction from the neighbors of the associated query compound. Multicore processing is used for SVM kernel parameter and hyper parameter optimization, which keeps runtime efficiently under control even for large sets of neighbors.

#### Applicability domains

Applicability domain estimation is a core module of the lazar algorithm, and is closely tied to the prediction algorithm, subject to the same validation procedures as predictions. Conceptually, the following factors affect the applicability domain of an individual prediction:

- Number of neighbors
- Similarities of neighbors
- Coherence of experimental data within neighbors

Consequently, a prediction based on a large number of neighbors with high similarity and concordant experimental data will be more reliable than a prediction based on a low number of neighbors with low similarity and contradictory experimental results. Hence, the *confidence* of the lazar algorithm is even more comprehensive than classical applicability domain approaches that only consider the feature value space, but not the coherence of the endpoint values.

More formally, the *confidence* of a prediction is defined by the mean neighbor similarity (see similarity indices for the different cases of neighbor similarity).

### IMPLEMENTATION

lazar is based on the OpenTox (Hardy et al., 2010) framework and consists of four main layers:

**Clients** Command line and graphical user interfaces using the ruby library.

**Ruby library** Ruby abstraction of the OpenTox REST API.

**Webservices** OpenTox compliant webservices for compounds, features, datasets, algorithms, models, validation, tasks.

**Backends** Special purpose backends for data storage (4store), authentication and authorization (OpenSSO), statistical computing (Rserve).

The main implementation language is Ruby. Computationally expensive parts are written in C/C++, while statistical computing is delegated to R. Both backends are dynamically loaded into Ruby via dynamic libraries and Ruby’s native language interface. Services communicate through the OpenTox REST API using Resource Description Framework (RDF) as the primary data exchange format. In depth discussion of implementation details can be found on the web at http://opentox.github.com.

### AVAILABILITY

A web interface for lazar is freely accessible from http://lazar.in-silico.ch. Public OpenTox compliant REST webservices exist at the URIs

- http://webservices.in-silico.ch/compound
- http://webservices.in-silico.ch/dataset
- http://webservices.in-silico.ch/algorithm
- http://webservices.in-silico.ch/model
- http://webservices.in-silico.ch/task

Source code has been published at Github^7^ under the GPL3 license. Ruby Gems for client and server libraries, webservices, and applications are hosted at http://gemcutter.org. Pre-installed and configured virtual appliances with commercial support can be obtained from *in silico* toxicology gmbh.

## EXPERIMENTS

During lazar development we have performed a large number of validation experiments to investigate various variants of the overall algorithm. As it is beyond the scope of a single paper to present all of them even in condensed form, we focus here on a few results which could be interesting for a larger community and justify the selection of lazar algorithms. For further reference, very detailed and up-to-date validation reports for all lazar models can be obtained from the lazar website at http://lazar.in-silico.ch. For the purpose, of this overview we have selected two example datasets, one for classification and one for regression (numerical predictions). Experiments include 10-fold cross-validation, and the creation of a validation report.

### CLASSIFICATION

For substructure-based models, we have shown that substantial improvements may be achieved by weighting each descriptor with its association to the endpoint (Maunz and Helma, 2008). For example, in the case of the fathead minnow acute toxicity dataset, the *p*-values were employed as weights in a kernel-based approach. The effects were twofold:

- A substantially higher fraction of molecules could be predicted, compared to the same setting without weighting.
- The predictive performance increased.

This indicates the utility of *p*-values to identify relevant descriptors, in that they are able to extract some relevant descriptors, and “mute” a large fraction of irrelevant descriptors, that would otherwise outweigh the former, simply because there are so many of them. We refer the reader to our earlier work (Maunz and Helma, 2008) for details. In the present implementation of lazar, *p*-value weighting is implemented by using a cutoff in the substructure mining step (see Substructure Mining).

The lazar algorithm with BBRC descriptors was applied to the Kazius/Bursi mutagenicity dataset (Kazius et al., 2005) using a 10-fold cross-validation. For each training fold, substructures were mined and a lazar model was built and subsequently applied to the corresponding test fold. Any instance was represented in bit vector form (fingerprints), where each index represents presence or absence of the corresponding descriptor. Weighted majority voting was used for prediction. The validation results are shown in **Tables 1** and **2**. Note that the given statistics neglect prediction confidences – higher accuracies can be achieved by setting a cutoff for acceptable confidences, albeit at the cost of obtaining fewer predictions.

**Table 1:** Validation statistics for the Kazius/Bursi dataset.

Num instances	4068
Num unpredicted	11
Accuracy	0.746
Area under roc	0.830
F measure	0.778
True positive rate	0.785
True negative rate	0.696
Positive predictive value	0.770
Negative predictive value	0.714

**Table 2:** Confusion table for the Kazius/Bursi dataset.

		Actual		Total
		Active	Inactive
Predicted	Active	1799	537	2336
	Inactive	492	1229	1721
Total		2291	1766	

**FIGURE 3:**
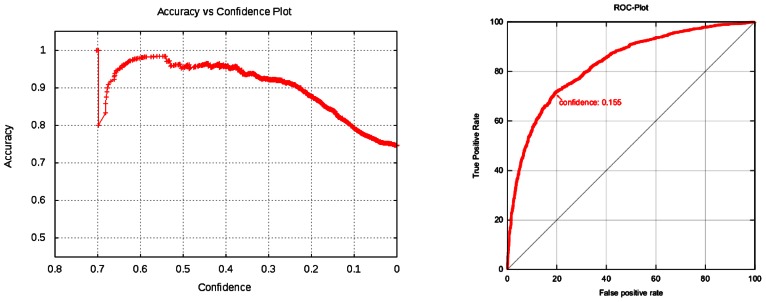
**Kazius/Bursi *Salmonella* mutagenicity dataset: total accuracy (left) and the class specific accuracies (right)**. The left plot shows that model accuracy decreases with decreasing confidence (variability at the left hand side of the plot can be ignored, because they are artifacts from small sample sizes). Note that prediction confidences are not probabilities or any statistical measure of model performance. These values can be obtained from plots in the validation reports, by identifying the confidence value on the *x*-axis and looking up the corresponding value (e.g., accuracy or *R*-Square) on the *y*-axis.

**Figure 3** plots total accuracy (left) and the class specific accuracies (right).

### REGRESSION

The fathead minnow acute toxicity dataset (Russom et al., 1997) was modeled using physico-chemical descriptors. As the computation of these descriptors is independent of the endpoint variable (unsupervised), the features can be computed prior to cross-validation. In contrast, supervised feature computation (like e.g., discriminative graph mining) has to be applied to each training fold to avoid information leakage. Any instance was represented in numeric vector form, where each index represented the corresponding descriptor value. Support vector regression was used, where for each prediction a dedicated SVM model was built on the neighbors. The parameters of the radial basis function kernel have been optimized using a grid-search with different parameter values. In more detail, the SVM was trained on a 8 x 8 grid for the cost parameter C and hyper parameter μ.

**FIGURE 4:**
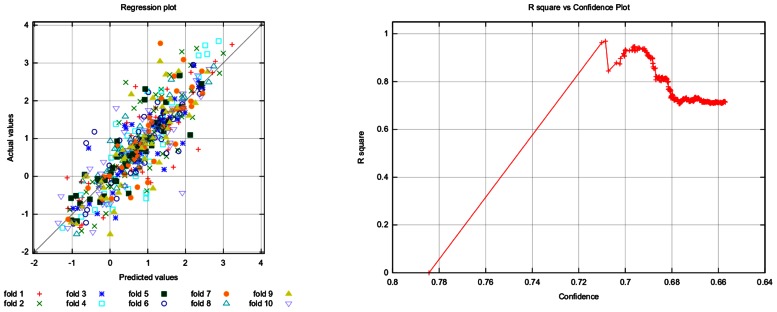
**Fathead minnow acute toxicity dataset: actual against predicted values (left) and *R*-squared against confidence (right)**. The left plot shows the correlation of model prediction and actual values. The plot on the right shows that the model performance decreases with decreasing confidence (see description of **Figure 3**).

**Table 3** provides common regression performance statistics, **Figure 4** plots actual against predicted values (left) and *R*-squared against confidence (right).

**Table 3:** Validation statistics for the fathead minnow dataset.

Num instances	535
Num unpredicted	76
Root mean squared error	0.586
Mean absolute error	0.428
*R*-squared	0.714
Sample correlation coefficient	0.846
Concordance correlation coefficient	0.833

**Figures 3** and **4** are excerpts of detailed validation reports from http://lazar.in-silico.ch that include the following information:

- Cross-validation statistics
- Confusion matrix (classification only)
- Plots: pairs of confidence vs. cross-validation statistics, ROC (classification), Scatterplot (Regression)
- Cross-validation statistics per fold
- All single predictions from all folds: 2D-structure image of compound, actual value, predicted value, confidence

## DISCUSSION

It is beyond the scope of this manuscript to present detailed validation results of all currently implemented lazar models. Detailed and up-to-date validation reports can be retrieved from the lazar websitehttp://lazar.in-silico.ch, and new regression models will be discussed in greater detail in a forthcoming publication. Instead, we will present a brief comparison of the lazar models from the Section “Experiments,” compare results from the literature, and discuss the consequences of the modular lazar design.

### MODEL PERFORMANCE

Comparisons with competing models from the literature are always difficult, because of different training sets, validation schemes, and performance estimates. To enable unbiased comparisons, we provide detailed validation reports, including not only all commonly used statistical performance indicators together with graphs, but also results for all training/test set splits, as well as tables of all validation instances with predicted and measured values, and applicability domain estimates^8^.

For the Kazius/Bursi mutagenicity data set, lazar made predictions for 4057 of the total 4068 compounds, only 11 compounds were outside of the applicability domain. Its AUC value of 0.83 ranks with the generic machine learning methods in the comparative study by Hansen et al. (2009), with AUC values between 0.79 and 0.86. It shows that these methods are clearly superior to the commercial systems DEREK and MultiCASE on this dataset. However, the authors point out the need for specific absorption rate (SAR) information, i.e., “interpretable structural information” on mutagenicity prediction, which generic machine learning methods do not provide. It should be pointed out that lazar provides both, predictive performance *and* detailed SAR information with every single prediction, among others all the substructures (here: BBRC descriptors) that were used to represent query compound and neighbors, as well as the neighbors themselves.

For fathead minnow acute toxicity, lazar predicted 535 of the total 611 compounds, which is comparable to the 555 in the study by In et al. (2012). In contrast to their approach, however, lazar determined the domain of applicability domain autonomously. Moreover, the lazar *R*-squared values, obtained by pooling the results from 10-fold cross-validation, are also substantially higher than their values (ranging between 0.553 and 0.632). They were obtained by a single train/test split, which can be considered less reliable. The *R*-squared values are also higher than the values in the overview for which they gathered results from the literature.

### APPLICABILITY DOMAINS

In contrast to generic machine learning methods, applicability domains are tightly integrated with the lazar framework, in that any prediction is associated with a confidence value. Cumulative plots of confidence and accuracy for the experiments discussed above are depicted in **Figures 3** and **4**. These figures document that the confidence value provides meaningful information, as the model accuracy decreases with decreasing confidence.

### MECHANISTIC INTERPRETATION

**FIGURE 5:**
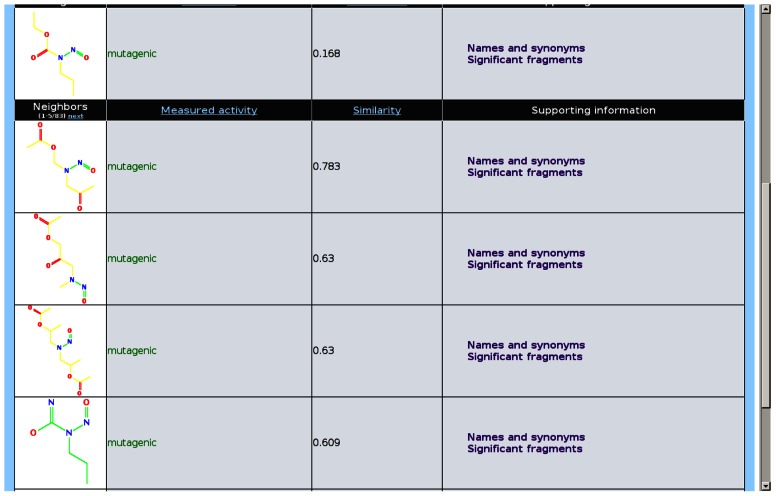
**lazar prediction example for *Salmonella* mutagenicity (Kazius/Bursi datset)**.

lazar intends to present the rationales for each prediction in a form that is understandable for toxicological experts without a background in machine learning and statistics. For this purpose, the following information is displayed graphically in the web interface (**Figure 5**):

- Neighbors that have been used for creating the local QSAR model, together with a graphical display of their structures, activity specific similarities, and experimental measurements
- Activating and deactivating fragments are highlighted in the query compound
- Definitions for domain specific terms can be obtained by following links in the web interface

By providing such detailed information we want to ensure that predictions are critically examined by toxicologists. Information about possible mechanisms can be obtained from neighbors (which are assumed to act by similar mechanisms as the query compound) and by the structural alerts used to determine activity specific similarities. In the present version of the web interface this information has to be retrieved manually, but we plan to add further visualization and search components (e.g., for obtaining and comparing pathway information of neighbors) in the future.

### LIMITATIONS

It is important to remember that lazar predictions are based on statistical criteria alone, without any explicit consideration of chemical or biological knowledge. This implies that lazar capabilities depend – like any other data driven approach – on size, composition, and quality of the training data. Large and reliable datasets with a good coverage of the chemical space will lead to more accurate predictions and a broader applicability domain than models based on small and unreliable datasets. Coherent endpoint values of similar compounds in the training dataset also increase the applicability domain of our approach. The quality of an individual prediction will depend also on the proximity of the query compound to the training data, which is represented by the confidence index.

One particular problem can arise when the query structure contains biologically active substructures that are not represented in sufficient number in the training set. In this case they cannot be evaluated statistically and will be classified as “inert” by the similarity calculation algorithm, which may lead to incorrect predictions. As it is impossible to compute such constraints automatically, a toxicological interpretation of lazar results is essential. For example, if a toxicologist discovers that a confirmed biologically active substructure is not present in the model, or that neighbors act by different mechanisms, it is better to discard the prediction than to trust it blindly.

### MODULAR DESIGN AND INTERACTION WITH THE SEMANTIC WEB

The modular structure of the lazar framework and its integration with the semantic web enables possibilities that go far beyond the currently implemented lazar prediction models. With the integration in the OpenTox framework, a researcher can freely combine algorithms for

- descriptor calculation (or use measured properties, e.g., from high throughput screening)
- descriptor selection
- similarity calculation
- model building

and validate the resulting model objectively with the OpenTox validation service. We are currently working on the development of nanoQSAR models that incorporate the behavior of engineered nanoparticles, as well as on predicting affected pathways within the lazar framework.

Currently, all major open source chemoinformatics and machine learning algorithms are supported by wrappers for OpenBabel, CDK, JoeLib, Weka, and R libraries, and the integration of newly developed algorithms is straightforward through OpenTox algorithm web services. The OpenTox API also allows the easy integration of lazar models into third party applications and frameworks like Bioclipse, Taverna, or Knime. lazar can also interact with external data sources (e.g., the Ambit database; Jeliazkova and Jeliazkov, 2011) and ontologies through the OpenTox API and data model. The integration of ontologies offers interesting possibilities that go far beyond simple QSAR model building, for example for the identification of adverse outcome pathways (Organisation for Economic Co-operation and Development [OECD], 2004a), supporting a more mechanistically oriented risk assessment procedure.

## CONCLUSION

lazar is a flexible modular framework for developing predictive toxicology models with a strong focus on the transparency and interpretability of predictions. Currently implemented lazar models perform competitively with the best results reported in the literature.

While the first principle (*a defined endpoint*) of the OECD principles for QSAR validation (Organisation for Economic Co-operation and Development [OECD], 2004b) cannot be supported directly by a computational framework, lazar clearly complies with the remaining principles (*an unambiguous algorithm, a defined domain of applicability, appropriate measures of goodness-of-fit*, robustness and predictivity, a mechanistic interpretation, if possible).

For future developments, lazar provides well established and tested algorithms, semantic web aware web services, and language bindings, which can serve as building blocks for new algorithms and applications. We hope that these facilities will speed up the development cycle of future predictive toxicology applications, and will ultimately lead to improved and more relevant applications in this area.

## Conflict of Interest Statement

The authors declare that the research was conducted in the absence of any commercial or financial relationships that could be construed as a potential conflict of interest.

## Footnotes

1 We have submitted a separate paper on this subject on this topic, for this reason we will cover validation only superficially in this manuscript.

2 http://github.com/opentox/algorithm

3 Expert knowledge, encoded in software is frequently used in predictive toxicology. Such expert systems build QSAR generalizations from individual chemicals to chemical classes based on prior knowledge, heuristics, expert judgment, and chemical and biological mechanism considerations. A prominent example is DEREK, sold by Lhasa Ltd. In systematic assessments of predictive power, such as the *Predictive Toxicology Evaluation*, however, expert systems have been performing rather badly, compared to statistical models (Srinivasan et al., 1997). A reason for their remarkable spread despite this crucial deficiency may be that their logic closely mimics the line of argumentation of chemical experts, which may provide an intuitive familiarity and seeming plausibility.

4 This is more or less the reverse procedure as in traditional hypothesis driven experimental science, where a scientist starts with a hypothesis, designs and conducts experiments and uses statistics to (in)validate hypothesis.">This is more or less the reverse procedure as in traditional hypothesis driven experimental science, where a scientist starts with a hypothesis, designs and conducts experiments and uses statistics to (in)validate hypothesis.

5 We plan a tighter integration of ontologies without compromising the statistical foundation of lazar in the near future.

6 Popular tools are e.g., DEREK, Toxtree (Patlewicz et al., 2008), or the OECD toolbox (available at http://www.oecd.org/env/ehs/risk-assessment/theoecdqsartoolbox.htm

7 http://github.com/opentox

08 http://lazar.in-silico.ch
